# Publisher Correction: Semaphorin 3A causes immune suppression by inducing cytoskeletal paralysis in tumour-specific CD8^+^ T cells

**DOI:** 10.1038/s41467-024-47775-7

**Published:** 2024-04-24

**Authors:** Mike B. Barnkob, Yale S. Michaels, Violaine André, Philip S. Macklin, Uzi Gileadi, Salvatore Valvo, Margarida Rei, Corinna Kulicke, Ji-Li Chen, Vitul Jain, Victoria K. Woodcock, Huw Colin-York, Andreas V. Hadjinicolaou, Youxin Kong, Viveka Mayya, Julie M. Mazet, Gracie-Jennah Mead, Joshua A. Bull, Pramila Rijal, Christopher W. Pugh, Alain R. Townsend, Audrey Gérard, Lars R. Olsen, Marco Fritzsche, Tudor A. Fulga, Michael L. Dustin, E. Yvonne Jones, Vincenzo Cerundolo

**Affiliations:** 1 MRC Human Immunology Unit, MRC Weatherall Institute of Molecular Medicine, University of Oxford, Headley Way, Oxford, OX3 9DS UK; 2 MRC Weatherall Institute of Molecular Medicine, University of Oxford, Headley Way, Oxford, OX3 9DS UK; 3 Nuffield Department of Medicine, University of Oxford, Nuffield Department of Medicine Research Building, Roosevelt Drive, Oxford, OX3 7FZ UK; 4 Kennedy Institute of Rheumatology, University of Oxford, Roosevelt Dr, Oxford, OX3 7FY UK; 5 Division of Structural Biology, Wellcome Centre for Human Genetics, University of Oxford, Roosevelt Drive, Oxford, OX3 7BN UK; 6 Wolfson Centre for Mathematical Biology, Mathematical Institute, University of Oxford, Radcliffe Observatory Quarter, Woodstock Road, Oxford, OX2 6GG UK; 7 Department of Health Technology, Technical University of Denmark, Ørsteds Plads, Building 345C, 2800 Kgs Lyngby, Denmark; 8 Present Address: Centre for Cellular Immunotherapy of Haematological Cancer Odense (CITCO), Department of Clinical Immunology, Odense University Hospital, University of Southern Denmark, Odense, Denmark; 9 Present Address: Paul Albrechtsen Research Institute, CancerCare Manitoba, 675 Mcdermot Ave, Winnipeg, MB R3E 0V9 Canada; 10 Present Address: Department of Biochemistry and Medical Genetics, Rady Faculty of Health Sciences, University of Manitoba, Bannatyne Ave, Winnipeg, MB R3E 3N4 Canada; 11 Present Address: Instituto de Medicina Molecular João Lobo Antunes, Faculdade de Medicina, Universidade de Lisboa, Lisbon, Portugal; 12 Present Address: Pulmonary and Critical Care Medicine, Oregon Health and Science University, Portland, OR US; 13 Present Address: Division of Gastroenterology & Hepatology, Department of Medicine, Cambridge University Hospitals, University of Cambridge, Cambridge, England; 14 Present Address: Early Cancer Institute, Department of Oncology, University of Cambridge, Cambridge, England

**Keywords:** Tumour immunology, Immune evasion, Cytotoxic T cells, Lymphocyte differentiation

Correction to: *Nature Communications* 10.1038/s41467-024-47424-z, published online 12 April 2024

In this article the title was incorrectly written as ‘Semmaphorin 3A causes immune suppression by inducing cytoskeletal paralysis in tumour-specific CD8+ T cells’ but should have been ‘Semaphorin 3A causes immune suppression by inducing cytoskeletal paralysis in tumour-specific CD8+ T cells’. The original article has been corrected.

