# Supplementary material for: Semaphorin 3A causes immune suppression by inducing cytoskeletal paralysis in tumour-specific CD8+ T cells
Source: Nat Commun. 2024 Apr 12;15:3173. doi: 10.1038/s41467-024-47424-z (PMC11017241; doi:10.1038/s41467-024-47424-z)
Supplement: Supplementary file 1 — Supplementary Information [file 41467_2024_47424_MOESM1_ESM.pdf]

Supplementary Information

Semaphorin 3A causes immune suppression by inducing cytoskeletal paralysis in tumour-specific CD8+ T cells

Mike B Barnkob[1,*], Yale S Michaels[2], Violaine André[1], Philip S Macklin[3], Uzi Gileadi[1], Salvatore Valvo[4], Margarida Rei[1], Corinna Kulicke[1], Ji-Li Chen[1], Vitul Jain[5], Victoria K Woodcock[1], Huw Colin-York[1], Andreas V Hadjinicolaou[1], Youxin Kong[5], Viveka Mayya[4], Julie M Mazet[4], Gracie-Jennah Mead[4], Joshua A Bull[6], Pramila Rijal[1], Christopher W Pugh[3], Alain R Townsend[1], Audrey Gérard[4], Lars R Olsen[7], Marco Fritzsche[1,4], Tudor A Fulga[2], Michael L Dustin[4], E Yvonne Jones[5,*], Vincenzo Cerundolo[1]

[1] MRC Human Immunology Unit, MRC Weatherall Institute of Molecular Medicine, University of Oxford, Headley Way, Oxford OX3 9DS, UK.
[2] MRC Weatherall Institute of Molecular Medicine, University of Oxford, Headley Way, Oxford OX3 9DS, UK.
[3] Nuffield Department of Medicine, University of Oxford, Nuffield Department of Medicine Research Building, Roosevelt Drive, Oxford OX3 7FZ, UK.
[4] Kennedy Institute of Rheumatology, University of Oxford, Roosevelt Dr, Oxford OX3 7FY, UK.
[5] Division of Structural Biology, Wellcome Centre for Human Genetics, University of Oxford, Roosevelt Drive, Oxford, OX3 7BN, UK.
[6] Wolfson Centre for Mathematical Biology, Mathematical Institute, University of Oxford, Radcliffe Observatory Quarter, Woodstock Road, Oxford, OX2 6GG, UK.

Correspondence: *E. Yvonne Jones (E.Y.J), The Wellcome Centre for Human Genetics, Roosevelt Drive, Oxford, OX3 7BN, e-mail: yvonne@strubi.ox.ac.uk, phone: +44 (0)1865 287 546 and Mike B Barnkob (M.B.B.), Department of Clinical Immunology, Odense University Hospital, J.B. Winsløv Vej 4, 5000 Odense C, Denmark, e-mail: mike.bogetofte.barnkob@rsyd.dk, phone: +45 27216890.

Supplementary Figure 1

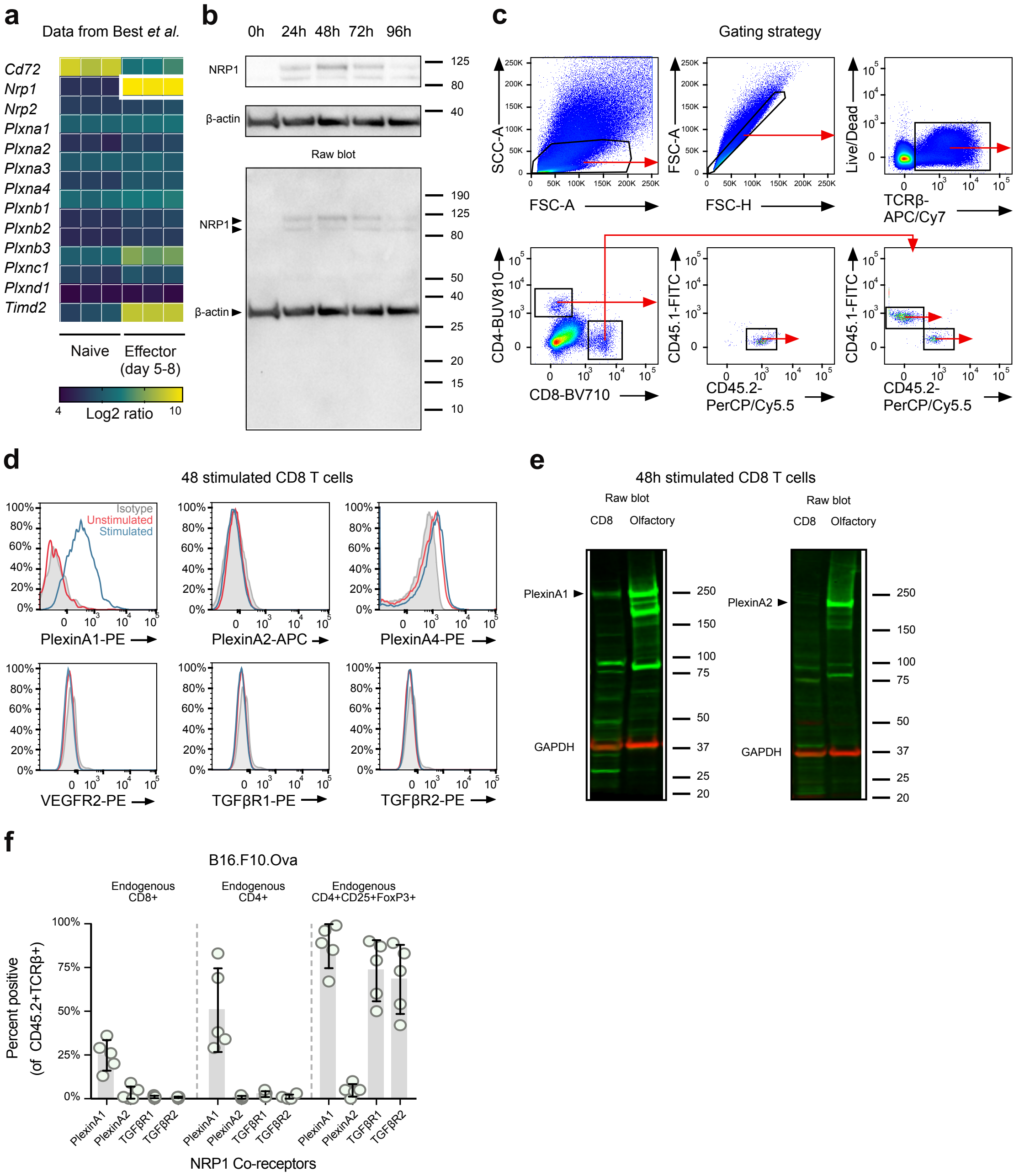

Supplementary Figure 1. Relates to Figure 1. NRP1 is expressed on CD8+ T cells. A. Heatmap of transcript levels of known semaphorin receptors on naïve and effector OT-I T cells following infection with vaccinia-OVA. Data from Best et al. 2013 (30). **B**. Western blot showing NRP1 upregulation in OT-I T cells following stimulation with SIINFEKL. Experiment performed once. **C**. Gating strategy for Figure 1F-G. **D**. Flow cytometric analysis of Plexin-A1, Plexin-A2, Plexin-A4, VEGFR2, TGFβR1 and TGFβR2 on unstimulated and 48 hour SIINFEKL stimulated OT-I T cells. Experiment representative of three independent

experiments. **E**. Western blots showing expression of Plexin-A1 (left, green) and Plexin-A2 (right, green) in 48 hour stimulated OT-I T cells and olfactory lobe (positive control for SEMA3A expression). Loading control GAPDH shown in red. Experiment performed once. **F**. Flow cytometric analysis of Plexin-A1, Plexin-A2, TGFβR1 and TGFβR2 expression on OT-I T cells and endogenous $CD8^+$ TILs, 11 days after adoptive transfer of OT-I T cells in antigen-expressing tumor (B16.F10.Ova) (n=5 mice). Experiment performed once. Error bars indicate standard deviation. Source data are provided as a Source Data file.

Supplementary Figure 2

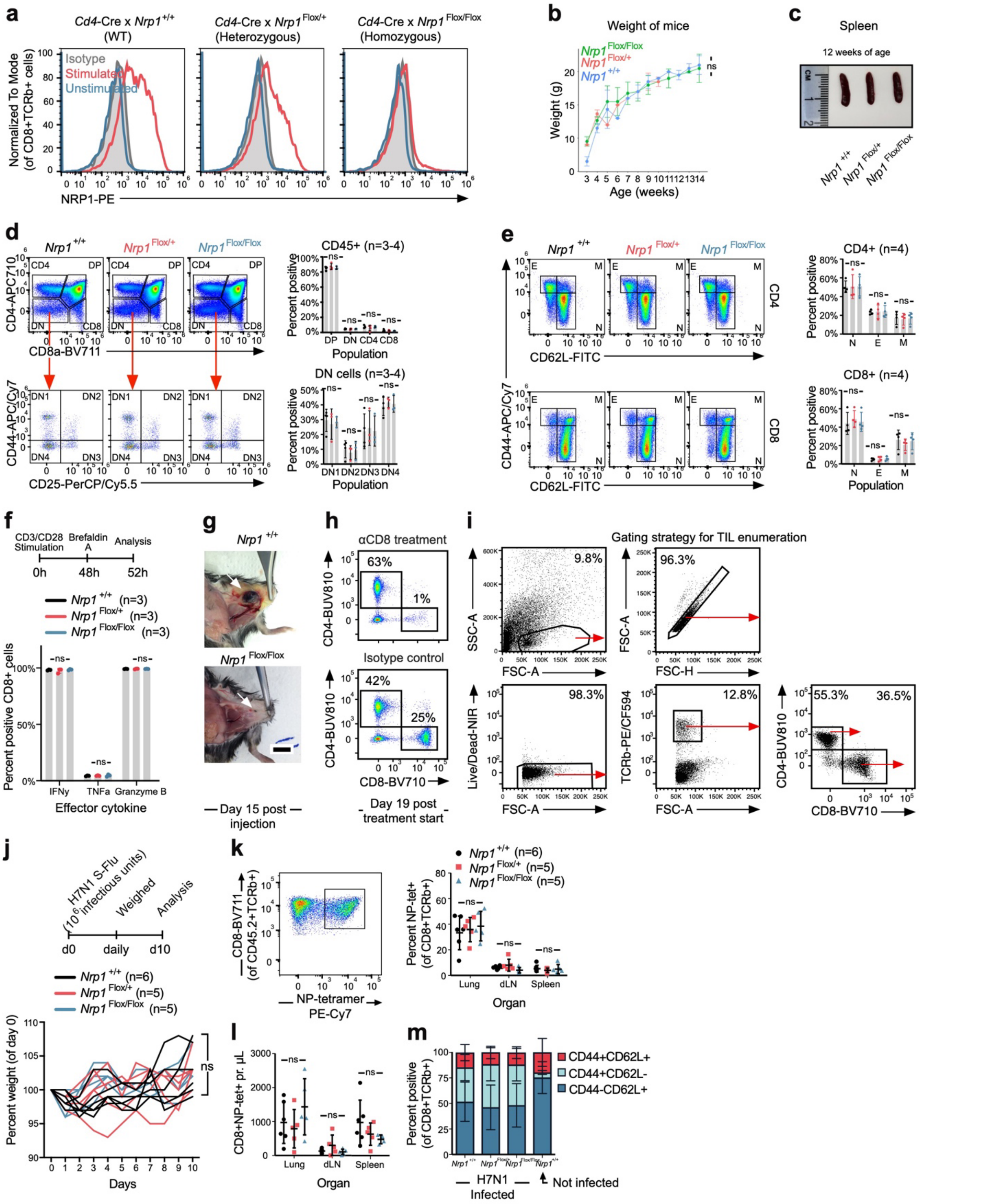

Supplementary Figure 2. Relates to Figure 2. Characterization of Cd4^{Cre} Nrp1$^{+/+}$ mice.

A. Flow cytometric analysis of naïve or CD3/CD28 stimulated splenocytes from either *Cd4^{Cre} Nrp1$^{+/+}$*, *Nrp1*$^{Flox/+}$ or *Nrp1*$^{Flox/Flox}$ mice. Cells are gated on CD8 and TCRβ. Experiment

performed independently three times. **B**. The weights of female littermates (n=17 mice) were followed for 12 weeks and revealed no difference between genotypes. Data indicate mean ± standard deviation. ns = not significant, by two-way ANOVA. **C**. The size of spleens from female littermate mice of different genotypes at 12 weeks of age. **D**. Representative plots showing the distribution of double negative, double positive, CD4 and CD8 positive thymocytes (upper panel, left) and DN1, DN2, DN3 and DN4 populations (lower panel, left) in $Cd4^{Cre}$ $Nrp1^{+/+}$, $Nrp1^{Flox/+}$ or $Nrp1^{Flox/Flox}$ mice (n=3-4 mice per genotype) as analyzed by flow cytometry. Cells are gated on CD45.2. Quantification of cell populations in different genotypes (upper and lower histograms, right). Experiment performed once. **E**. Representative plots showing T cell effector populations in splenic $CD4^+$ (top) and $CD8^+$ (bottom) T cells. Cells are gated on CD45.2 and TCRβ (n=4 mice per genotype). Data combined from two independent experiments. **F**. Cytokine production following *ex vivo* stimulation by CD3/CD28 beads in $Cd4^{Cre}$ $Nrp1^{+/+}$, $Nrp1^{Flox/+}$ or $Nrp1^{Flox/Flox}$ mice (n=3 mice). Experimental design (upper panel). Quantification of IFNγ, TNFα and Granzyme B by intracellular staining (lower panel). Cells are gated on TCRβ and CD8 (n=3 mice per genotype). Experiment repeated twice. **G**. Representative image of B16.F10 tumors 15 days post-injection in $Cd4^{Cre}$ $Nrp1^{+/+}$ (upper image) and $Nrp1^{Flox/Flox}$ (lower image) mice. Arrows indicates tumors. Scale bar indicates 10 mm. **H**. Representative flow cytometric analysis of peripheral blood in mice treated with either aCD8 antibodies (upper scatterplot) or isotype control (lower scatterplot). **I**. Gating strategy used for flow cytometric analysis of TIL enumeration in mice. **J**. Weight of mice following H7N1 S-Flu infection. Experimental design (upper panel). Weight of mice, normalized to day 0 of individual mouse weight (lower panel). Experiment performed once (n=5-6 mice per genotype). **K**. Analysis of H7N1 S-Flu-specific T cells 10 days post-infection. Example $H\text{-}2D^B$-NP tetramer staining in lung of infected mouse (left figure). Quantification of $H\text{-}2D^B$-NP tetramer positive $CD8^+$ T cells in lung, dLN and spleen (right figure). Cells are gated on CD45.2, TCRβ and CD8 (n=5-6 mice per genotype). Experiment performed once. **L**. Quantification of total number of infiltrating H-$2D^B$-NP tetramer positive $CD8^+$ in lung, dLN and spleen 10 days post-infection. Experiment performed once. **M**. Analysis of effector subpopulations in lung 10 days post-infection in different genotypes of mice (n=5-6 mice per genotype). Error bars indicate standard deviation. ns = not significant, by two-way ANOVA (D, E, F, J, K). Abbreviations: dLN,

draining lymph node. DN, double negative. E, effector T cells. N, naïve T cells. M, memory T cells. TIL, tumor-infiltrating leukocytes. Source data are provided as a Source Data file.

Supplementary Figure 3

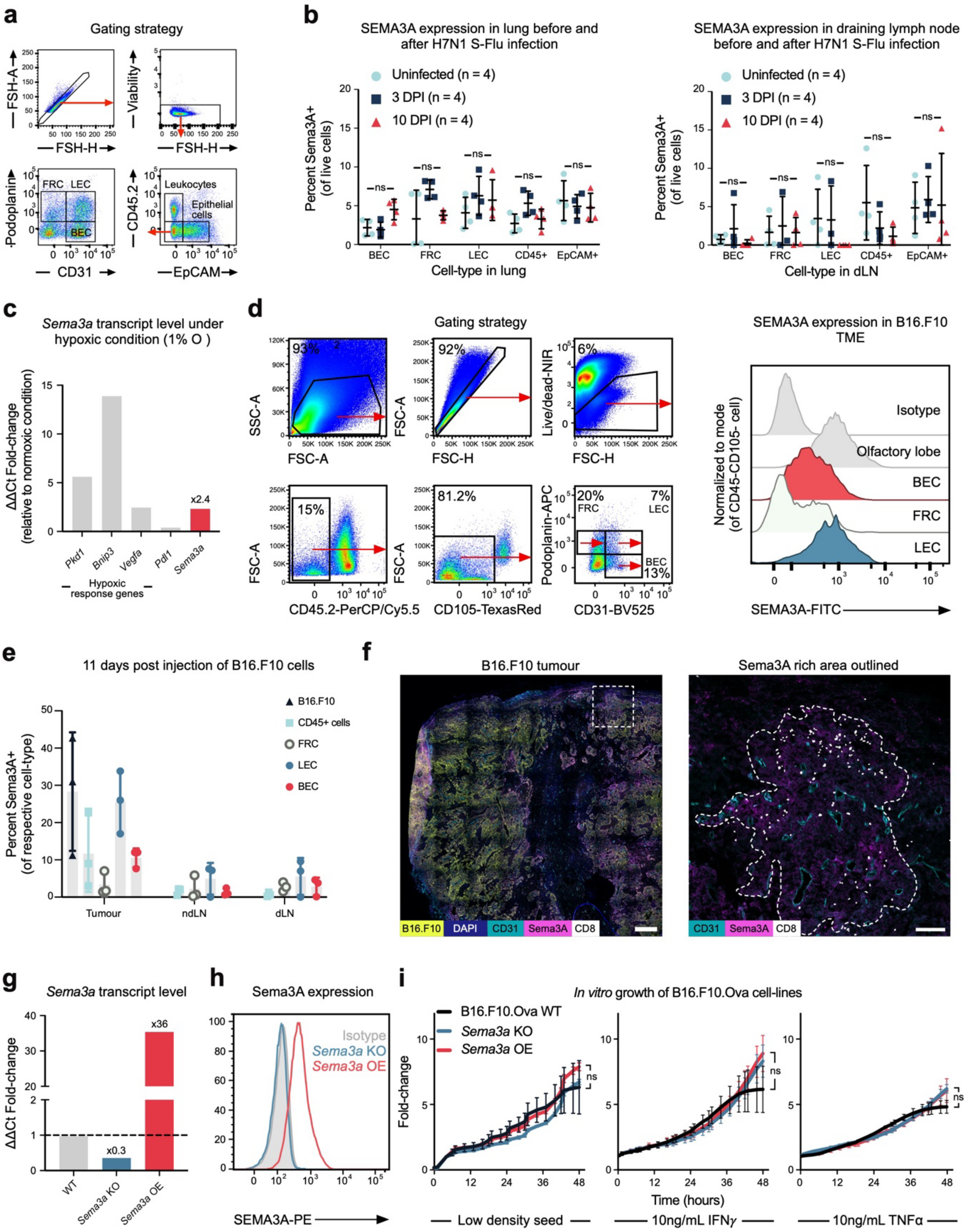

Supplementary Figure 3. Relates to Figure 2. Sema3A expression in lungs and B16.F10 tumours. A. Gating strategy for analyzing SEMA3A expression among leukocytes,

epithelial and endothelial cells in lung and dLN. **B**. Quantification of SEMA3A positive cells in different cell populations in uninfected (n=4) and infected mice at 3 days DPI (n=4 mice) and 10 DPI (n=4 mice) in lung (left panel) or dLN (right panel). *Cd4*^{Cre} *Nrp1*$^{\text{Flox/Flox}}$ mice used. Data combined from two independent experiments. **C**. Quantification of *Pkd1*, *Bnip3*, *Vegfa*, *Pdl1* and *Sema3a* mRNA level following 24 hour culture in 1% O2 chamber. **D**. Gating strategy (left) and representative histograms (right) analyzing SEMA3A positive cell populations in B16.F10 tumors 11 days post-injection. Olfactory lobe is used as a positive control for SEMA3A expression. **E**. Quantification of SEMA3A positive cells in same experiment as in (D) in tumor, dLN and ndLN. **F**. Immunofluorescent image of B16.F10 tumour 15 days post injection, scalebar = 500 uM (left). Dashed region is shown at higher magnification, scalebar = 100 uM (right). **G**. RT-qPCR analysis show downregulation and upregulation of *Sema3a* mRNA in *Sema3a* KO and OE cell lines, respectively (normalized to *Hprt*). Experiment performed once. **H**. Intracellular staining shows no detectable expression of SEMA3A in *Sema3a* KO cells and expression in *Sema3a* OE cells. Experiment performed once, at low seeding density. **I**. Growth of WT, *Sema3a* KO and *Sema3a* OE B16.F10.Ova cell lines in normal, IFNγ or TNFα rich media. Experiment performed once. Error bars indicate standard deviation. ns = not significant, by two-way ANOVA (B, I). Abbreviations: BEC, blood endothelial cells. dLN, draining lymph node. DPI, days post-infection. FRC, fibroblastic reticular cells. KO, knockout. LEC, lymphatic endothelial cells. ndLN, non-draining lymph node. OE, overexpressing. TME, tumor microenvironment. WT, wild-type. Source data are provided as a Source Data file.

Supplementary Figure 4

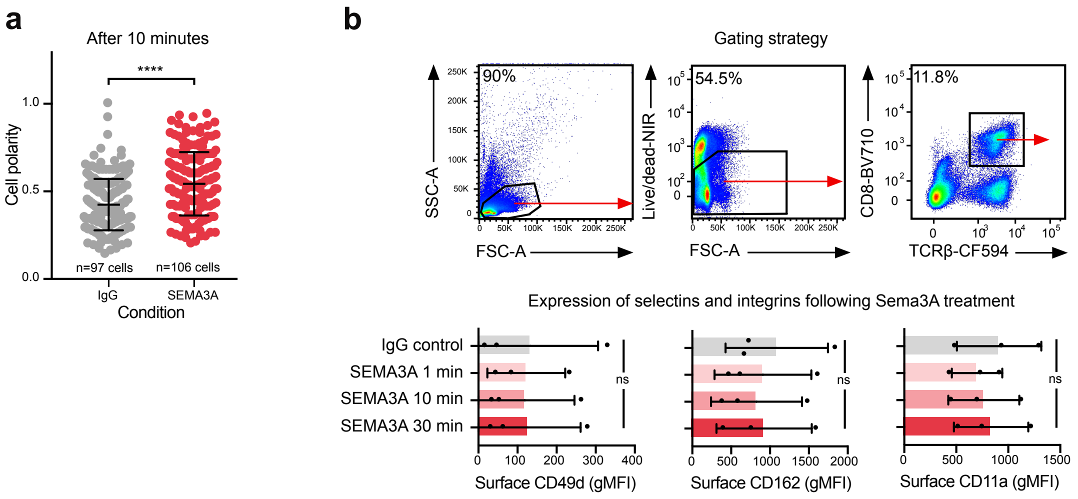

Supplementary Figure 4. Relates to Figure 3. Sema3A's effect on selectins and integrins. A. Relative frequency of cell polarity of 48 hour stimulated OT-I T cells treated with IgG or SEMA3A$_{S-P}$. A polarity of 1 indicates a shape of a perfect circle, 0 a rectangular shape. Experiment repeated three times. Error bars indicate standard deviation. **** = $P < 0.0001$, by Student's t-test. **B**. Gating strategy for analyzing 48 hour stimulated OT-I splenocytes treated with SEMA3A$_{S-P}$ (top). Bar graphs of gMFI of CD49d, CD162 and CD11a following SEMA3A$_{S-P}$ treatment at indicated times. ns = not significant, by Kruskal-Wallis test. Source data are provided as a Source Data file.

Supplementary Figure 5

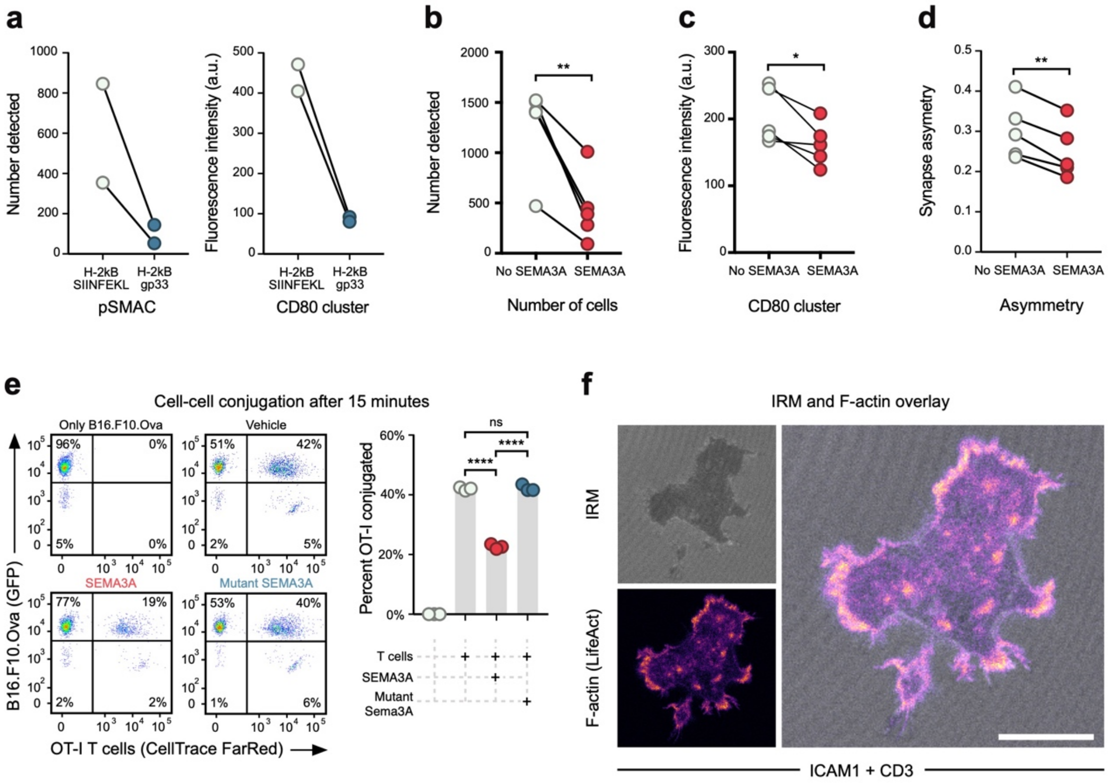

Supplementary Figure 5. Relates to Figure 4. Sema3A's effect on immune synapse and cell-cell binding of T-cells. A. Quantification of pSMAC (left) and CD80 clustering (right) in immunological synapses of 48 hour stimulated OT-I T cells when presented with a relevant (H-2Kb-SIINFEKL) or irrelevant (H-2Kb-gp33) MHC monomer loaded onto the bilayer. Data combined from two independent experiments. **B**. Quantification of 48 hour stimulated OT-I T cells detected in high-throughput assay with or without SEMA3A$_{S\text{-}P\text{-}I}$ pre-treatment. Data combined from five independent experiments. ** = P = 0.0069, by paired t-test.

C. Conducted as in B. Fluorescence intensity of CD80 signal introduced by 48 hour stimulated OT-I T cells in high-throughput assay with or without SEMA3A$_{S\text{-}P\text{-}I}$ pre-treatment. * = P < 0.0272, by paired t-test. **D**. Conducted as in B. Analysis of radial symmetry of synapses in OT-I T cells in high-throughput assay with or without SEMA3A$_{S\text{-}P\text{-}I}$ pre-treatment. Asymmetry of the synapse is quantified as the distance between the centroids of the CD80 cluster and that of the pSMAC relative to the diameter of the cell. ** = P < 0.0013, by paired t-test. **E**. Gating strategy and representative images showing number of B16.F10.Ova cells and T cells as either singlets or doublets under four different conditions:

cancer cells alone, with normal media, media with $SEMA3A_{S-P}$ or with mutant SEMA3A (left). Quantification of three biological replicates, showing approximately 50% reduction in cell-cell conjugation when SEMA3A is present (right). Data representative of three independent experiments. **** = $P < 0.0001$, ns = not significant, by two-way ANOVA. Gray bars indicate mean. **F**. Images from live-cell imaging of OT-I × LifeAct T cells showing concordance between IRM shadow and F-actin signal. Scale bar indicates 10 µm. Abbreviations: IRM, interference reflection microscopy. pSMAC, peripheral supramolecular activation cluster. Source data are provided as a Source Data file.

Supplementary Figure 6

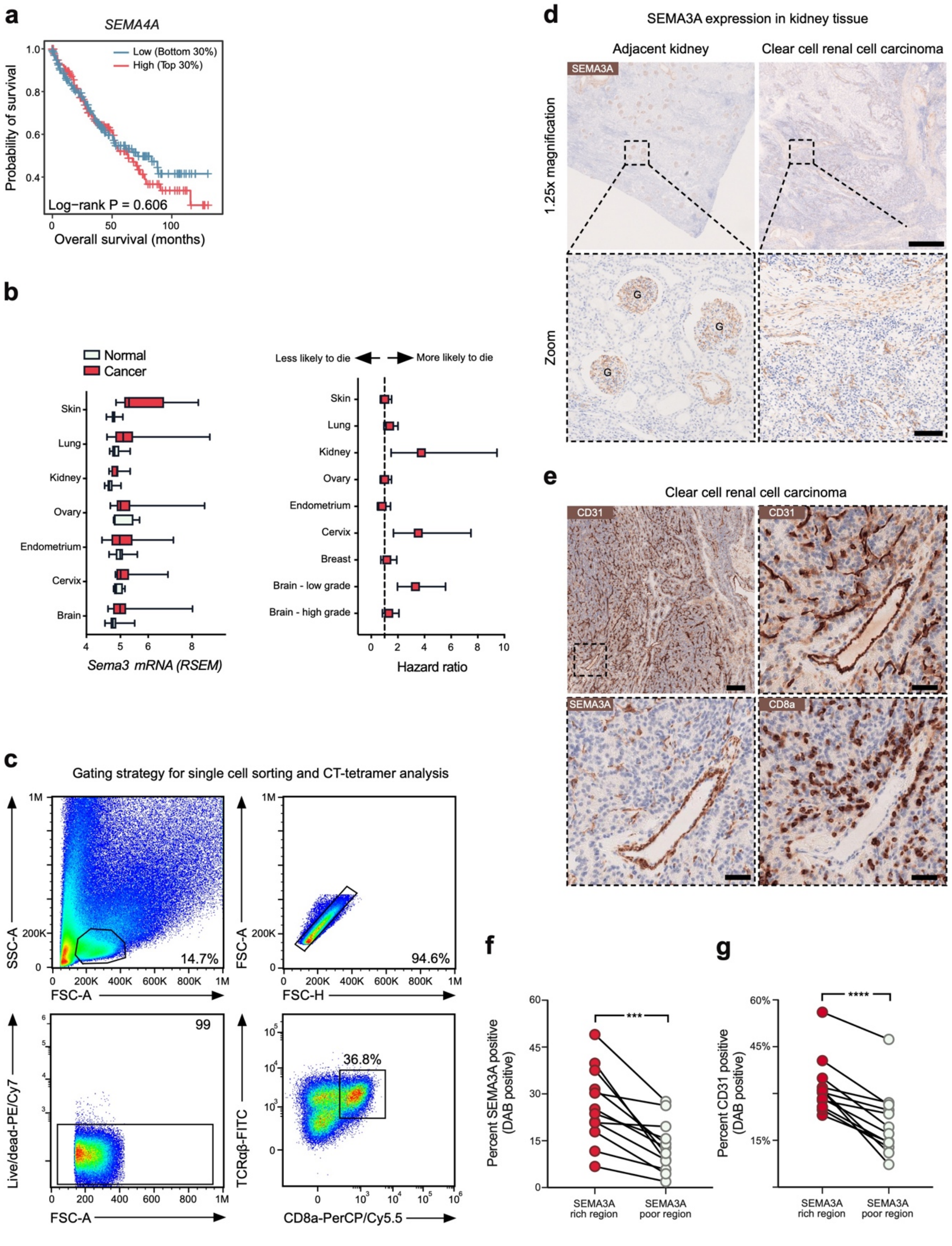

Supplementary Figure 6. Relates to Figure 6. Sema3A expression in ccRCC. A, Correlation of *SEMA4A* mRNA level with survival of ccRCC patients. **B**. Analysis of SEMA3A mRNA level in normal tissue and cancer tissue (left). Hazard ratio for survival comparing

high-expressors with low-expressors (right). Error bars indicate standard deviation. **C.** Gating strategy used to identify CD8+ T-cells for sorting and tetramer analysis. **D**. SEMA3A expression in tumor-adjacent and tumor tissue of ccRCC patient. G indicates kidney glomeruli. Scalebars indicate 1000 µm (upper row) and 100 µm (lower row). **E**. Serial sections from ccRCC tumor stained for CD31, SEMA3A and CD8a. Dashed box in upper left image indicates the region depicted at higher magnification in the three other images. Scale bar indicates 200 µm (upper left) and 50 µm (other images). **F**. Expression of CD31 in SEMA3A rich and poor regions. *** = $P < 0.0005$, by paired t-test. **G**. Expression of SEMA3A in selected SEMA3A rich and poor regions. **** = $P < 0.0001$, by paired t-test. Abbreviations: ccRCC, clear cell renal cell carcinoma. DAB, 3,3'-Diaminobenzidine. Source data are provided as a Source Data file.

Supplementary Table 1

Baseline characteristics of patient cohort.		
Characteristics	Number (range)	Percent
**Age (years)**		
Mean	64.4 (42-86)	
**Gender**		
Male	13	56.5%
Female	10	43.5%
**Tumor grade (ISUP)**		
1	0	0.0%
2	2	8.7%
3	12	52.2%
4	6	26.1%
N/A	3	13.0%
**Tumor location**		
Right	14	60.9%
Left	9	39.1%
**Type of surgery**		
Radical nephrectomy	17	73.9%
Partial nephrectomy	6	26.1%
**Tumor stage**		
pT1a	1	4.5%
pT1b	5	22.7%
pT2a	1	4.5%
pT2b	0	0.0%
pT3a	14	63.6%
pT3b	0	0.0%
pT3c	1	4.5%
pT4	0	0.0%
N/A	1	4.5%
