# Supplementary material for: Semaphorin 3A causes immune suppression by inducing cytoskeletal paralysis in tumour-specific CD8+ T cells
Source: Nat Commun. 2024 Apr 12;15:3173. doi: 10.1038/s41467-024-47424-z (PMC11017241; doi:10.1038/s41467-024-47424-z)
Supplement: Supplementary file 3 — Description of Additional Supplementary Files [file 41467_2024_47424_MOESM3_ESM.pdf]

Description of Additional Supplementary Files

Supplementary Movie 1

Related to Figure 4A-B. Time-lapse movie using interference reflection microscopy (IRM) of 48 hour activated CD8+ T cells immediately after being dropped on an activating surface displaying immobilized ICAM-1 and anti-CD3 antibodies. Media consists of T-cell media with 5uM control IgG. 1 frame per second. Scalebar = 10 uM.

Supplementary Movie 2

Related to Figure 4A-B. Time-lapse movie using interference reflection microscopy (IRM) of 48 hour actiated CD8+ T cells immediately after being dropped on an activating surface displaying immobilized ICAM-1 and anti-CD3 antibodies. Media consists of T-cell media with 5uM Sema3AS-P. 1 frame per second. Scalebar = 10 uM.

Supplementary Movie 3

Related to Figure 4C. Time-lapse movie using total internal reflection fluorescence (TIRF) microscopy of 48 hour activated OT-I T cells, pre-treated for 30 min with 5uM fluorescently-labelled SEMA3AS-P-I, before added to on supported lipid bilayer presenting H2Kb-SIINFEKL, CD80, and ICAM-1. SEMA3AS-P-I visualized as magenta; CD80 as green; ICAM-1 as blue. OT-I T cells binding Sema3A (magenta) do not form classical immunological synapses, while T-cells not binding Sema3A do. 1 frame per 20 seconds. Scalebar = 10 uM.

Supplementary Movie 4

Related to figure 5B-C. Time-lapse movie using confocal microscopy of 48 hour activated LifeAct-OT-I T cells before and after addition of Sema3A to media (after 5 minutes, frame 31). Left side is confocal image, while right side is the bright-field of the same frames. 1 frame per 10 seconds. Scalebar = 10 uM.

Supplementary Movie 5

Related to Figure 5H. Time-lapse movie using IRM of 48 hour activated OT-I T cells at three stages: in T-cell media (first three minutes, before addition of other proteins); with addition of Sema3AS-P (for three minutes); and with the further addition of Blebbistatin (for final three minutes). Movie shows the T cell stopping its behavior following addition of Sema3A, and then resuming it, following addition of Blebbistatin. 1 frame per second. Scalebar = 10 uM.

Supplementary Movie 6

Related to Figure 5I. Time-lapse movie using IRM of 48 hour activated OT-I T cells at three stages: in T-cell media (first three minutes, before addition of other proteins); with addition of Blebbistatin (for three minutes); and with the further addition of Sema3AS-P (for final three minutes). Movie shows normal behavior in the T cell following Blebbistatin addition, and after a few seconds effect of Sema3A, continued normal active behavior after Sema3A addition. 1 frame per second. Scalebar = 10 uM.
