# Supplementary material for: Semaphorin 3A causes immune suppression by inducing cytoskeletal paralysis in tumour-specific CD8+ T cells
Source: Nat Commun. 2024 Apr 12;15:3173. doi: 10.1038/s41467-024-47424-z (PMC11017241; doi:10.1038/s41467-024-47424-z)
Supplement: Supplementary file 10 — Reporting Summary [file 41467_2024_47424_MOESM10_ESM.pdf]

nature portfolio

Corresponding author(s): Mike Bogetofte Barnkob and E Yvonne Jones

Last updated by author(s): 19/02/2024

Reporting Summary

Nature Portfolio wishes to improve the reproducibility of the work that we publish. This form provides structure for consistency and transparency in reporting. For further information on Nature Portfolio policies, see our Editorial Policies and the Editorial Policy Checklist.

Statistics

For all statistical analyses, confirm that the following items are present in the figure legend, table legend, main text, or Methods section.

n/a	Confirmed	
[ ]	[x]	The exact sample size (*n*) for each experimental group/condition, given as a discrete number and unit of measurement
[ ]	[x]	A statement on whether measurements were taken from distinct samples or whether the same sample was measured repeatedly
[ ]	[x]	The statistical test(s) used AND whether they are one- or two-sided<br>*Only common tests should be described solely by name; describe more complex techniques in the Methods section.*
[x]	[ ]	A description of all covariates tested
[x]	[ ]	A description of any assumptions or corrections, such as tests of normality and adjustment for multiple comparisons
[ ]	[x]	A full description of the statistical parameters including central tendency (e.g. means) or other basic estimates (e.g. regression coefficient) AND variation (e.g. standard deviation) or associated estimates of uncertainty (e.g. confidence intervals)
[ ]	[x]	For null hypothesis testing, the test statistic (e.g. *F*, *t*, *r*) with confidence intervals, effect sizes, degrees of freedom and *P* value noted<br>*Give P values as exact values whenever suitable.*
[x]	[ ]	For Bayesian analysis, information on the choice of priors and Markov chain Monte Carlo settings
[x]	[ ]	For hierarchical and complex designs, identification of the appropriate level for tests and full reporting of outcomes
[x]	[ ]	Estimates of effect sizes (e.g. Cohen's *d*, Pearson's *r*), indicating how they were calculated

Our web collection on statistics for biologists contains articles on many of the points above.

Software and code

Policy information about availability of computer code

Data collection	Flow cytometers: Attune NxT (Life Technologies), LSR Fortessa X20 or X50 (BD Biosciences). FACS: SH800 cell sorter (SONY). Microscopes: DeltaVision Elite Live cell imaging microscope, Zeiss LSM 780 or 880 confocal, FluoView FV1200 confocal (Olympus), InCell 6000 (Cytiva), NanoZoomer S210 digital slide (Hamamatsu), and Odyssey Near-Infrared imaging system (LI-COR). Sequencer: MiSeqV2 (Illumina). RT-qPCR: QuantStudio7 qRT-PCR (Life Technologies).
Data analysis	Excel 2016 (Microsoft); Prism software version 8 (GraphPad); FlowJo version 10 (BD Biosciences); R version 3.4, with the following packages: tidyverse, ggplot2, affy, limma, VDJtools; MATLAB (MathWorks); Fiji/ImageJ, with the following packages: Trackmate; Webtools: CRISPResso, TIMER. Code for analysis of immunological synapse avaiable here: doi.org/10.5281/zenodo.10522993. R Code for spider plots of cell movements, TRBV analysis, and analysis of GSE15907 data is available upon request. Analysis scripts for analysis of ccRCC samples have been described in Bull JA, et al. "Combining multiple spatial statistics enhances the description of immune cell localisation within tumours". Sci Rep. 2020;10(1):18624 and are available at doi.org/10.5281/zenodo.10625167.

For manuscripts utilizing custom algorithms or software that are central to the research but not yet described in published literature, software must be made available to editors and reviewers. We strongly encourage code deposition in a community repository (e.g. GitHub). See the Nature Portfolio guidelines for submitting code & software for further information.

Data

Policy information about availability of data

All manuscripts must include a data availability statement. This statement should provide the following information, where applicable:

- Accession codes, unique identifiers, or web links for publicly available datasets
- A description of any restrictions on data availability
- For clinical datasets or third party data, please ensure that the statement adheres to our policy

The CDR3 and TRBV usage data generated in this study have been deposited in the Sequence Read Archive under accession code PRJNA1075074 (https://www.ncbi.nlm.nih.gov/bioproject/PRJNA1075074). Analysis of survival data in ccRCC patients was done on The Cancer Genome Atlas (TCGA) data using the TIMER website: http://timer.cistrome.org/ (Taiwen Li et al, "TIMER: A web server for comprehensive analysis of tumor-infiltrating immune cells.", Cancer Research. 2017). Analysis of SEMA3A receptors/ligands was done on data from “Immunological Genome Project data Phase 1” (series accession: GSE15907), available at: https://www.ncbi.nlm.nih.gov/geo/query/acc.cgi?acc=GSE15907, as described in "Analysis of publicly available transcriptional data" in the Method section.

Research involving human participants, their data, or biological material

Policy information about studies with human participants or human data. See also policy information about sex, gender (identity/presentation), and sexual orientation and race, ethnicity and racism.

Reporting on sex and gender	For ccRCC patients, 13 were male and 10 were female (23 total). Sex was not a determining factor for including patients. Sex was determined based on sex at birth. Disaggregated gender data is not available. No sex- or gender-based analysis were performed as sex is not expected to affect major differences in the immune subsets examined here.
Reporting on race, ethnicity, or other socially relevant groupings	N/A
Population characteristics	Age and tumour grade was collected. Population characteristics were not considered in the study design and analysis.
Recruitment	Patients scheduled for surgery for renal cancer at the Oxford University Hospitals NHS Foundation Trust were recruited. Patients were only included after written consent was obtained, according to institutional guidelines and after the project was approved by the Oxfordshire Research Ethics Committee C.
Ethics oversight	Oxfordshire Research Ethics Committee C.

Note that full information on the approval of the study protocol must also be provided in the manuscript.

Field-specific reporting

Please select the one below that is the best fit for your research. If you are not sure, read the appropriate sections before making your selection.

☒ Life sciences ☐ Behavioural & social sciences ☐ Ecological, evolutionary & environmental sciences

For a reference copy of the document with all sections, see nature.com/documents/nr-reporting-summary-flat.pdf

Life sciences study design

All studies must disclose on these points even when the disclosure is negative.

Sample size	No sample size calculations were performed, but was based on our previous experience in dealing with the tumour models used here. Based on these experiences, 5-10 mice were used per experiment. At least two independent replicates performed, unless stated otherwise in figure legend/methods section. For analysis involving human primary cells, at least three donors were used to verify reproducibility of results.
Data exclusions	No data points were excluded from analysis.
Replication	Replication number are defined in the figure legends. For animal studies, experiments were performed at least twice, unless otherwise stated in the figure legends. Cell-culture experiments were repeated at least three times, unless otherwise stated in figure legends. All separate experiments yielded comparable trends and results.
Randomization	For all experiments, conditions and animals were kept as closely to each other as possible to minimize any potential co-variates (e.g. all were females, genetically similar, and treated at the same age intervals, at 6-10 weeks of age). Animals were then randomly selected from cages when allocated to experimental group. For experiments involving tumour-bearing mice, each mouse was randomized before treatment by allocation into specific cages.
Blinding	Investigators were not blinded for experimental group when performing animal experiments as these were tagged for identification purposes. Data analysis was setup on one sample, typically control samples, and copied to all other samples which ensures blinding to individual groups and samples. Analysis of ccRCC IHC was done blinded.

Reporting for specific materials, systems and methods

We require information from authors about some types of materials, experimental systems and methods used in many studies. Here, indicate whether each material, system or method listed is relevant to your study. If you are not sure if a list item applies to your research, read the appropriate section before selecting a response.

Materials & experimental systems

n/a	Involved in the study
	x Antibodies
	x Eukaryotic cell lines
x	Palaeontology and archaeology
	x Animals and other organisms
x	Clinical data
x	Dual use research of concern
x	Plants

Methods

n/a	Involved in the study
x	ChIP-seq
	x Flow cytometry
x	MRI-based neuroimaging

Antibodies

Antibodies used

For flow cytometry:

Antigen - Fluorochrome - Reactivity - Clone - Provider - Dilution.
CD3e BV650 Mouse SK7 BioLegend 344872 1:100
CD4 APC710 Mouse GK1.5 Tonbo Bio 20-0041 1:200
CD4 BUV810 Mouse GK1.5 BD 553730 1:100
CD8a BV711 Mouse 53-6.7 BioLegend 100748 1:100
CD8a PerCP/ Cy5.5 Human HIT8a BioLegend 300924 1:100
CD11a PE Mouse M17/4 BioLegend 101107 1:100
CD11b APC Mouse M1/70 BioLegend 101212 1:100
CD19 BV435 Mouse 6D5 BioLegend 115506 1:100
CD24 APC710 Mouse M1/69 BD 562349 1:100
CD25 PerCP/Cy5.5 Mouse 3C7 BioLegend 101912 1:200
CD31 BV510 Mouse MEC13.3 BD 563089 1:100
CD44 APC/Cy7 Mouse IM7 BioLegend 103028 1:100
CD44 PE/Cy7 Mouse IM7 BioLegend 103030 1:100
CD45 APC Human HI30 BioLegend 304012 1:200
CD45.1 FITC Mouse A20 eBioScience 11-0453-82 1:100
CD45.2 PerCP/Cy5.5 Mouse 104 BioLegend 109828 1:100
CD49d BUV395 Mouse 9C10 BD 740219 1:100
CD62L BV610 Mouse Mel-14 BioLegend 104408 1:100
CD62L FITC Mouse Mel-14 BioLegend 104406 1:100
CD69 BUV737 Mouse H1.2F3 BD 612793 1:100
CD103 PE/Cy7 Mouse 2E7 BioLegend 121426 1:100
CD105 PE/CF594 Mouse MJ7/18 BioLegend 562762 1:100
CD162 BV421 Mouse 2PH1 BD 562807 1:100
EpCAM APC/Cy7 Mouse G8.8 BioLegend 118218 1:100
F4/80 BV610 Mouse BM8 BioLegend 123110 1:100
FoxP3 BV421 Mouse MF-14 BioLegend 126419 1:50
IFNy PE Mouse XMG1.2 BioLegend 505808 1:100
Granzyme B FITC Mouse GB11 BioLegend 515403 1:100
Ly6C BV780 Mouse HK1.4 BioLegend 128016 1:100
MHC-II PerCP/Cy5.5 Mouse AF6-120.1 BioLegend 116416 1:100
NRP1 BV421 Mouse 3E12 BioLegend 145209 1:100
NRP1 PE Mouse 3E12 BioLegend 145204 1:100
NRP1 BV421 Human 12C2 BioLegend 354514 1:100
HLA-A2/ SLLMWITQV APC Human In-house 1:100
H-2DB-NP PE/Cy7 Mouse In-house 1:100
H2-Kb/SIINFEKL APC Mouse In-house 1:100
PD-1 eFluor610 Human eBioJ105 eBioScience 61-2799-42 1:100
PD-1 APC Human EH12.2H7 BioLegend 329908 1:100
PlexinA1 PE Mouse 408305 R&D Systems FAB4309P 1:50
PlexinA2 APC Mouse 583603 R&D Systems FAB5486A 1:50
PlexinA4 PE Mouse ab39350 Abcam ab39350 1:100
Podoplanin APC Mouse 8.1.1 BioLegend 127410 1:100
TCRab FITC Human IP26 BioLegend 306706 1:100

TCRb APC/Cy7 Mouse H57-597 BioLegend 109220 1:100
TCRb PE-CF594 Mouse H57-597 BD 562841 1:100
TGFbRI PE Mouse 141231 R&D Systems FAB5871P 1:50
TGFbRII PE Mouse FAB532P R&D Systems FAB532P 1:50
TNFa PerCP/Cy5.5 Mouse MP6-XT22 BioLegend 506322 1:100
Sema3A AF488 Mouse 215803 R&D Systems IC1250G 1:100
Sema3A PE Mouse 215803 R&D Systems IC1250P 1:100
VEGFR2 PE Mouse Avas12 BioLegend 136404 1:100

For western blot:

Antigen - Reactivity - Clone - Provider - Catolog
NRP1 Mouse EPR3113 Abcam ab184783
Plexin A1 Mouse AF4309 R&D Systems AF4309
Plexin A2 Mouse AF5486 R&D Systems AF5486
GAPDH Mouse 6C5 Santa Cruz sc-32233
β-Actin Mouse 13E5 Cell Signaling Technology 4970S

For immunofluorescent and immunohistochemistry images:

Antigen - Reactivity - Clone - Provider - Catolog - Concentration
CD8 Human C8/144B Agilent Technologies M710301-2 1:100
CD8-AF647 Mouse EPR21769 Abcam ab237365 1:100
CD31 Human JC70A Agilent Technologies M082301-2 1:800
CD31 Mouse MEC13.3 BioLegend 102501 1:100
Goat Anti-Rat-AF555 Rat A-21434 Invitrogen A21434 1:300
Sema3A Human EPR19367 Abcam ab199475 1:4000
Sema3A-AF488 Mouse 215803 R&D Systems IC1250G 1:100

Other:

Antigen - Reactivity - Clone - Provider - Catolog - Concentration
CD3 Mouse 145-2C11 BioLegender 100366 10 µg/mL
CD8 Mouse 2.43 BioXcell BE0061
IgG2b Mouse LTF-2 BioXcell BP0090
NRP1 Mouse AF566 R&D Systems AF566 5ug/mL

Validation

All antibodies were obtained from commercial vendors and selected based on specificity on descriptions and information provided in corresponding data sheets available and provided by the manufacturers. Commercial antibodies were validated by vendor according to industry standards (as inserted below). H2-Kb/SIINFEKL, H-2DB-NP and HLA-A2 CT-antigen tetramers were made in-house and tested and validated on positive and negative cells.

Dilution optimization was performed on splenocytes or immune cells isolated from tumours.

Biolegend (human and mouse) Flow Cytometry Reagents:-Specificity testing of 1-3 target cell types with either single- or multi-color analysis (including positive and negative cell types). Once specificity is confirmed, each new lot must perform with similar intensity to the in-date reference lot. Brightness (MFI) is evaluated from both positive and negative populations. Each lot product is validated by QC testing with a series of titration dilutions. https://www.biolegend.com/en-us/quality/quality-control

BioXCell: Advanced Binding Validation utilizes a library of recombinant proteins and bioassay expertise to validate that each lot of applicable InVivoPlusTM antibody binds strongly and specifically to its target antigen.

AbCam: When validating antibodies for flow cytometry, we are careful to optimize every step of the staining protocol, including fixation, permeabilization, and washing. To do this, our scientists review the available literature to understand which cell types and conditions are best suited to validate specific antibodies. We include relevant controls, routinely running unstained, positive, negative, isotype, viability, Fc-blocking, fluorescence minus one (FMO), and single-staining controls. For an FMO control, we stain all our samples with fluorescent conjugates except the one that is being tested. This shows the contribution of the other fluorescent conjugates in the signal of the unlabeled channel. This control is important for determining non-specific binding of an antibody.

R&D Systems: We recognize the need to enhance antibody validation for our customers. In addition to our current validation procedures, Novus will now implement several new methods for antibody validation, in accordance with recommendations instigated by the International Working Group for Antibody Validation (IWGAV). Genetic Strategy Validation- Expression of the target protein is compared before and after knockout or knockdown using CRISPR/CAS9 or siRNA/shRNA. If protein expression following knockout or knockdown is substantially reduced, then antibody specificity is ensured.
Orthogonal Validation- The target protein is examined with an antibody independent strategy and compared with results from an antibody-dependent strategy. A correlation between these two strategies indicates specificity between the antibody and its target protein.
Independent Antibody Validation- The data generated using several antibodies (ideally targeting different epitopes) in the same protein is compared (e.g. molecular weight and cellular localization). Consistent results imply antibody selectivity to the target protein.

Expression of Tagged Proteins Validation- A tagged protein is used as a standard for comparison in Western blotting and/or immunocytochemistry (ICC). For example, if the distribution of the tagged protein overlaps with the immunofluorescence signal, then antibody specificity is confirmed.
Biological Strategies Validation- These strategies use defined biological or chemical modulation of protein expression to demonstrate antibody specificity to the target protein. The data is compared across multiple cell lines including positive and negative expressing cells, and multiple species, if applicable.

Cell Signalling: To ensure our antibodies will work in your experiment, we adhere to the Hallmarks of Antibody Validation, six complementary strategies that can be used to determine the functionality, specificity, and sensitivity of an antibody in any given assay. CST adapted the work by Uhlen, et. al., ("A Proposal for Validation of Antibodies." Nature Methods (2016)) to build the Hallmarks of Antibody Validation, based on our decades of experience as an antibody manufacturer and our dedication to reproducible science.

BD: Antibody specificity BD Biosciences identifies key targets of interest in scientific research and develops its own specific antibodies or collaborates with top research scientists around the world to license their antibodies. We then transform these antibodies into flow cytometry reagents by conjugating them to a broad portfolio of high-performing dyes, including our vastly popular portfolio of BD Horizon BrilliantTM Dyes. A world-class team of research scientists helps ensure that these reagents work reliably and consistently for flow cytometry applications. The specificity is confirmed using multiple methodologies that may include a combination of flow cytometry, immunofluorescence, immunohistochemistry or western blot to test staining on a combination of primary cells, cell lines or transfectant models. All flow cytometry reagents are titrated on the relevant positive or negative cells. To save time and cell samples for researchers, test size reagents are bottled at an optimal concentration with the best signal-to-noise ratio on relevant models during the product development. To ensure consistent performance from lot-to-lot, each reagent is bottled to match the previous lot MFI. You can look up the Certificate of Analysis and the concentration of test-size human reagents from specific lots via the Concentration Lookup page or BD Regulatory Documents. Technical data sheets provide data generated on the relevant primary model at this optimal concentration based on a titration curve. QC data on any lot of reagent can be requested through ResearchApplications@bd.com. Quality control Our dedication to rigorous testing and high-quality control standards means that you can use our reagents in your research with the utmost confidence. All BD reagent facilities, including our California Design Center at San Diego, our manufacturing facilities in Tatabanya (Hungary) and San Diego (USA), and our California instrument facility (Manufacturing and Design Centers) at San Jose, are approved and registered according to the internationally defined ISO 9001 standard. Once our research and development (R&D) team completes evaluation of a new product, the developed process is transferred to our manufacturing teams, including Quality Control. Our manufacturing process adheres to standard operating procedures (SOPs) and guidelines, which are based on ISO requirements and are strictly followed, helping ensure that reagents provide consistent results to help give you assurance of experimental success and confidence in your research. Quality control testing of newly manufactured lots is performed side-by-side with a previously accepted lot as a control, helping to assure that performance of the new lot is both reliable and consistent. https://www.bdbiosciences.com/en-us/products/reagents/flow-cytometry-reagents/research-reagents/quality-and-reproducibility

Eukaryotic cell lines

Policy information about cell lines and Sex and Gender in Research

Cell line source(s)	B16-F10 was originally from ATCC (catalog CRL-6475), and modified in-house to express OVA and Sema3A, as indicated in the method section. LL-2 cell-lines was originally from ATCC (catalog CRL-1642) and a gift from Professor Christopher W Pugh (University of Oxford).
Authentication	Cell-lines were not authenticated following purchase from ATCC. OVA expression of B16-F10 cells have continously been confirmed using in vitro activation assays with transgenic T cells (OT-I). Cell-lines had consistent behavior both in vitro and in vivo over the time of this manuscript.
Mycoplasma contamination	Cells were tested every 2nd month for Mycoplasma and always found negative.
Commonly misidentified lines (See ICLAC register)	No commenly misidentified lines used.

Animals and other research organisms

Policy information about studies involving animals; ARRIVE guidelines recommended for reporting animal research, and Sex and Gender in Research

Laboratory animals	All animals used were mice on C57BL/6 background, female and between 6-12 weeks of age. The following strains were used: CD4Cre mice (Tg(Cd4-cre)1Cwi/BfluJ Jackson Lab, stock no. 017336), Nrp1 floxed mice (B6.129(SJL)-Nrp1tm2Ddg/J, Jackson Lab, stock no. 005247), LifeAct-eGFP mice (Riedl, J. et al. Lifeact mice for studying F-actin dynamics. Nat. Methods 7, 168–169 (2010).), CD45.1 mice (B6.SJL-PtprcaPepcb/BoyCrl, Charles Rivers Strain Code 494), OT-I mice (C57BL/6-Tg(TcraTcrb)1100Mjb/Crl, Charles Rivers Strain Code 642).<br><br>Animals were housed in dedidated facilities in individually ventilated cages (IVC) with corn cob bedding, ad libitum water and chew blocks. 12 hours of light/dark cycle with half an hour of dim light period was used. Appropriate environmental enrichment was provided. The temperature was maintained at 21 degrees +/- 2 and the humidity 55% +/- 10.
Wild animals	No wild animals used.
Reporting on sex	All tumour were grown in female mice. OT-I T-cells were collected from male mice.

Field-collected samples	No field-collected samples used.
Ethics oversight	All animal studies were carried out in accordance with Animals (Scientific Procedures) Act of 1987. The protocols are reviewed by local Veterinary Surgeon (Vet) and Named Animal Care and Welfare Officer (NACWO) at University of Oxford before being reviewed and approved by Animal Welfare and Ethical Review Body (AWERB) at University of Oxford/Oxfordshire.

Note that full information on the approval of the study protocol must also be provided in the manuscript.

Plants

Seed stocks	N/A
Novel plant genotypes	N/A
Authentication	N/A

Flow Cytometry

Plots

Confirm that:

- [x] The axis labels state the marker and fluorochrome used (e.g. CD4-FITC).
- [x] The axis scales are clearly visible. Include numbers along axes only for bottom left plot of group (a 'group' is an analysis of identical markers).
- [x] All plots are contour plots with outliers or pseudocolor plots.
- [x] A numerical value for number of cells or percentage (with statistics) is provided.

Methodology

Sample preparation	Cells from spleens were strained through a a 70 µm nylon mesh, washed and resuspended in media. Cells from B16.F10 and LL/2 tumours, lymph nodes, frontal cortex, lungs or thymus were cut into smaller pieces and incubated for 30 minutes with reagents from a tumour dissociation kit (130-096-730, Miltenyi Biotec), strained through a 70 µm nylon mesh, washed and resuspended in 100% Percoll solution (17-0891-01, GE Healthcare), and layered carefully on top of 3 mL of 80% and 40% Percoll solution. After 30 minutes at 2000g, cells at the 80-40% interphase were collected and stained.
Instrument	Attune NxT (Life Technologies), LSR Fortessa X20 or X50 (BD Biosciences), SH800 cell sorter (SONY).
Software	FlowJo version 10 (BD Biosciences)
Cell population abundance	>98%
Gating strategy	Gating strategies are shown in Extended Figure 1C, 2D, 2I, 3A, 3D, 4B, 6A, 6C.

- [x] Tick this box to confirm that a figure exemplifying the gating strategy is provided in the Supplementary Information.
